# ID 272 - Enoxaparina: da síntese de evidências ao protocolo clínico ampliado para profilaxia e tratamento de tromboembolismo venoso em gestantes e puérperas no estado da Bahia

**DOI:** 10.5327/2237-9622.2025.v34s1.272

**Published:** 2025-11-25

**Authors:** Carolina Santos Silva, Bárbara de Castro Santos Silva, Gleidson Cardoso Spínola, Manuela Fernandes de Almeida Mello, Rouseli Gonçalves de Menezes, Danielli Nunes de Oliveira Costa

**Keywords:** enoxaparina, tromboembolismo venoso, avalição de tecnologias em saúde, protocolos clínicos

## Abstract

**Introdução:**

As trombofilias são condições que podem aumentar o risco de trombose venosa (TEV). A prevenção do TEV está centrada na terapia com anticoagulantes para mulheres com fatores de risco adicionais à gestação. A enoxaparina é a anticoagulação de escolha. Muitas diretrizes concordam sobre a avaliação dos fatores de risco para TEV no início da gravidez, visto que mulheres com alto risco estão recomendadas à tromboprofilaxia, porém sem consenso sobre os grupos de maior risco de TEV e duração da tromboprofilaxia. O objetivo deste estudo foi avaliar eficácia, segurança e custos da enoxaparina para tromboprofilaxia em gestantes ou puérperas com risco para TEV, visando à ampliação das indicações previstas no Protoclo Clínico e Diretrizes Terapêuticas (PCDT) nacional vigente.

**Método:**

As buscas foram realizadas nas bases MEDLINE; LILACS e Embase no mês de janeiro de 2024. A estratégia combinou população-alvo, enoxaparina e heparinas de baixo peso molecular (HBPM); filtros para revisão sistemática (RS) com ou sem metanálise e publicações até cinco anos, sem restrição de idioma e do status da publicação. A elegibilidade dos estudos ocorreu em duas etapas por dois revisores e com discordâncias resolvidas por um terceiro revisor. Identificaram-se 349 publicações. Após exclusões, oito atenderam aos critérios de seleção. O impacto orçamentário usou valor e doses da enoxaparina, seu consumo hospitalar local e dados epidemiológicos. O Sistema de Apuração e Gestão de Custos do Sistema Único de Saúde (ApuraSUS) e o Epimed foram utilizados para cálculo do custo diário, médio e anual do internamento obstétrico de gestantes com TEV na Bahia.

**Resultados:**

Foram encontrados os seguintes resultados: redução de eventos tromboembólicos com HBPM; uma RS convergiu com o PCDT nacional em relação à necessidade de anticoagulação nos casos de TEV prévio; uma RS fez recomendações de profilaxia congruentes com as diretrizes do (RCOG) ao indicar diminuição de 84% dos eventos de TEV em gestantes de alto risco perinatal (história de TEV, hipertensão gestacional, diabetes, idade avançada, obesidade, parto múltiplo e tabagismo), porém sem estratificar os dados para as subpopulações; outra RS apresentou 78% de redução de TEV em puérperas que não fizeram anticoagulação no anteparto. Houve aumento de risco de eventos hemorrágicos nas gestantes com maior agravo (136%) na fertilização in vitro (FIV); redução de 42% no risco de aborto e em perdas gestacionais e aumento de 15% da taxa de nascidos vivos ; aumento importante de eventos adversos, principalmente cutâneos.

O custo médio da diária para internação de gestantes de alto risco em 2023 foi de R$ 732,14 (ApuraSUS). O custo anual médio evitável foi de R$ 1.388.137,44, usando estimativas da população-alvo do Epimed. O impacto orçamentário foi de R$ 945.362,97 no primeiro ano.

**Conclusão:**

Este estudo demonstrou benefício em gestantes de alto risco de TEV no perinatal e em puérperas sem anticoagulação no anteparto, mas obteve evidências científicas frágeis para ampliação do uso de enoxaparina, pois há escassez de publicações estratificando a população-alvo nos diversos níveis de risco para TEV e para o tipo de HBPM. Sem novidades nos desfechos de segurança em relação ao PCDT nacional. A análise econômica evidenciou alto custo médio das internações prolongadas de gestantes para uso de enoxaparina. Nesse contexto, a gestão da Secretaria de Saúde do Estado da Bahia (Sesab) por um protocolo estadual com ampliação das indicações de anticoagulação, observando: alta vulnerabilidade dessa população; qualificação da assistência, redução da ocupação de leitos e custos de internamentos; tentativa de prevenir morbimortalidade materna.

